# The Per2 Negative Feedback Loop Sets the Period in the Mammalian Circadian Clock Mechanism

**DOI:** 10.1371/journal.pcbi.0030242

**Published:** 2007-12-14

**Authors:** A. Katharina Wilkins, Paul I Barton, Bruce Tidor

**Affiliations:** 1 Department of Chemical Engineering, Massachusetts Institute of Technology, Cambridge, Massachusetts, United States of America; 2 Computer Science and Artificial Intelligence Laboratory, Massachusetts Institute of Technology, Cambridge, Massachusetts, United States of America; 3 Department of Biological Engineering, Massachusetts Institute of Technology, Cambridge, Massachusetts, United States of America; 4 Department of Electrical Engineering and Computer Science, Massachusetts Institute of Technology, Cambridge, Massachusetts, United States of America; University of Washington, United States of America

## Abstract

Processes that repeat in time, such as the cell cycle, the circadian rhythm, and seasonal variations, are prevalent in biology. Mathematical models can represent our knowledge of the underlying mechanisms, and numerical methods can then facilitate analysis, which forms the foundation for a more integrated understanding as well as for design and intervention. Here, the intracellular molecular network responsible for the mammalian circadian clock system was studied. A new formulation of detailed sensitivity analysis is introduced and applied to elucidate the influence of individual rate processes, represented through their parameters, on network functional characteristics. One of four negative feedback loops in the model, the Per2 loop, was uniquely identified as most responsible for setting the period of oscillation; none of the other feedback loops were found to play as substantial a role. The analysis further suggested that the activity of the kinases CK1δ and CK1ɛ were well placed within the network such that they could be instrumental in implementing short-term adjustments to the period in the circadian clock system. The numerical results reported here are supported by previously published experimental data.

## Author Summary

Network models of biological systems are appearing at an increasing rate. By encapsulating mechanistic detail of chemical and physical processes, mathematical models can successfully simulate and predict emergent network properties. However, methods are needed for analyzing the role played by individual biochemical steps in producing context-dependent system behavior, thereby linking individual molecular knowledge with network properties. Here, we apply sensitivity analysis to analyze mammalian circadian rhythms and find that a contiguous series of reactions in one of the four negative feedback loops carries primary responsibility for determining the intrinsic length of day. The key reactions, all involving the gene *per2* and its products, include Per2 mRNA export and degradation, and PER2 phosphorylation, transcription, and translation. Interestingly, mutations affecting PER2 phosphorylation have previously been linked to circadian disorders. The method may be generally applicable to probe structure–function relationships in biological networks.

## Introduction

The circadian clock is a well-studied oscillatory biological system. It is nearly ubiquitous in eukaryotes and is found in similar versions in very different organisms, from unicellular cyanobacteria through filamentous fungi and plants to mammals [1]. It provides a mechanism for adaptation to the changing environment following a 24 h cycle, by, for example, readying the organism in advance for the next event of the day. In addition to establishing periods of wakefulness and rest, the mammalian circadian clock regulates many bodily functions, such as renal and liver activity and the release of appropriate hormones at different times [2]. The circadian clock is the pacemaker that in its normal function is responsible for the impact of shift work and jet lag on alertness, behavior, and health, and whose misregulation plays a role in such disorders as familial advanced sleep phase syndrome (FASPS). In patients afflicted with FASPS, a shortened intrinsic period makes it difficult for affected individuals to have a normal work and social life. In addition to these more well-known effects, circadian rhythms also play a role in pathogenesis and can guide optimal treatment for diseases, including arthritis, asthma, cancer, cardiovascular disease, diabetes, duodenal ulcers, hypercholesterolemia, and seasonal affective disorder [3,4]. In many instances, circadian rhythms can be exploited to minimize dosage and side effects by timing appropriate therapies to the peak times of disease activity or symptoms, including pain [4]. A better understanding of the circadian clock and its workings might contribute to improved treatment of these disorders.

Current models of circadian clocks show behaviors consistent with known biology and anticipated from engineering principles, such as a persistence of the free running period (FRP) in the absence of a daily stimulus and the ability to entrain to periodic external signals [2]. In addition, the circadian clock, particularly that of organisms lacking temperature regulation, exhibits temperature compensation—the period of oscillation is insensitive to changes in the external temperature [2].

Despite detailed studies on the molecular as well as the systems level [5–7], open questions persist. Some can be addressed using mathematical analysis of the biological models, and examples from this class form the focus of the current work. Is there a difference in mechanism between phase advance and phase delay, as suggested by experimental observation that phase delay happens much more rapidly than phase advance [6]? Which input pathways could potentially play a role in managing such phase responses? Is the fact that the FRP of the human circadian clock is slightly larger than 24 h related to the difference in phase advance and delay?

As a first step toward answering such questions, which typically involve the simultaneous analysis of several network characteristics, we focus on the period-specific biochemical properties of the mammalian circadian network. We discuss which network structures are involved in setting the FRP, as revealed by detailed sensitivity analysis. The distribution of this responsibility within the network gives important clues toward a further understanding of the principles and concepts underlying network design. Intimately related to studying where in the network the FRP is regulated is the study of possible mechanisms present to modify the FRP temporarily or persistently, in order to accommodate external fluctuations. How flexibly can the system adjust to changing external situations, for example by undergoing phase shifts? A potential point of intervention for the short-term management of the FRP is suggested here.

The fundamental biochemical pathways involved in the clock systems of different eukaryotic species are well-known and have the same essential components. Negative feedback regulation of transcription is always present and often interlocked with positive feedback, thus increasing the complexity. Nuclear transport of transcriptional regulators is a central process in forming the feedback loops [2].

At the heart of the mammalian clock is the CLOCK protein (CLK), which acts together with BMAL1 in a heterodimeric complex (BCC). BCC is a transcriptional activator of the three *per* (period) homologues, two *cry* (cryptochrome) homologues, and the *rev-erbα* gene. REV-ERBα represses *bmal1* expression, and regulates *clock* and *cry1* expression [8]. Because *cry1* expression is lowered by REV-ERBα, and CRY1 is an inhibitor of the BCC, which activates *rev-erbα* expression, a positive feedback loop is formed. The PER proteins are phosphorylated by several isoforms of casein kinase 1 (CK1δ, CK1ɛ, and possibly others) in a complex manner that regulates their degradation and nuclear trafficking [9]. PER1 and PER2 can form stable complexes with the kinases and either CRY protein [2,10]. The PER proteins are rate-limiting for this step and necessary for the nuclear import of the complex, making them the shuttle for nuclear CRY proteins [11]. Nuclear CRY and PER proteins all have an inhibitory effect on the activity of the BCC, probably following different mechanisms [10], thereby closing four negative feedback loops affecting the expression of *cry* and *per* genes. PER2 plays a second role in the positive regulation of *bmal1* expression [12]. Of the *per* homologues, *per3* is the only one whose deletion hardly affects the rhythmicity of the system. While the roles of the two *cry* homologues appear similar and perhaps redundant, this is not so for the *per* homologues [13]. In humans, a single mutation in *per2* causes FASPS [14], and its loss causes arrhythmicity in mice [15,16]. The behavioral phenotypes of *per1* null mutant mice were similar to those of *per2* mutants; however, comparison of the molecular consequences of the mutations revealed significant differences between the two. Disruption of *per2* expression was reported to result in reduced transcription levels of other clock genes, whereas PER1 appears to act predominantly at the posttranscriptional level [16]. An overview of the molecular mechanism of the circadian clock is found in [2] and [12].

Several mathematical models of circadian clock systems in different organisms have been formulated in recent years ([7,17–21], among others), providing different levels of detail. The most detailed model of the mammalian circadian clock to date was published by Forger and Peskin in 2003 [18] and was used for the current study. It describes the mechanistic action of 73 species (proteins and mRNA, both in the cytosol and the nucleus) and uses 38 parameters to model their interactions. The mathematical form of the model is a system of ordinary differential equations (ODEs), and all reactions are modeled using mass action kinetics.

**Figure 1:**
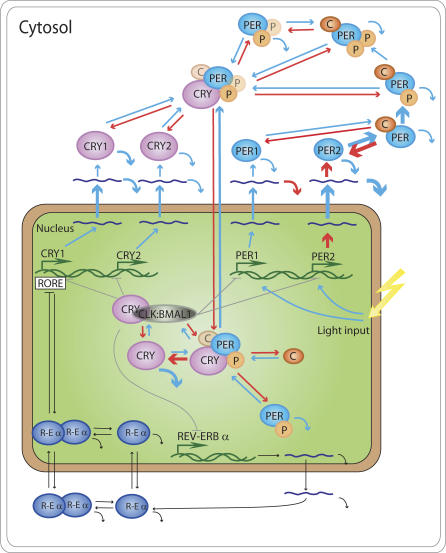
Graphical Representation of Results of Detailed Sensitivity Analysis Red, positive sensitivity; blue, negative sensitivity; black, zero sensitivity; gray, not modeled explicitly; thick arrow, large magnitude; thin arrow, small magnitude; curved arrow, degradation; R-Eα, REV-ERBα; RORE, target sequence for retinoic acid–related orphan receptor R-Eα on Cry1 promoter; P, phosphorylated; C, CK1. The CRY-CLK:BMAL1 complex does not inhibit transcription, but rather diverts the activator CLK:BMAL1 from the transcription initiation site. The effect of light input is modeled as an increase in *per1* and *per2* transcription, identical for both. In the interest of visualization, only Per2 pertinent sensitivities are represented where one arrow represents multiple processes. In those cases, the Per2-related sensitivities were always significantly larger in magnitude than any other sensitivities.

As shown in Figure 1, the model includes separate feedback loops for two homologues each of Cry and Per. The third Per homologue, Per3, is not included, as its role is not well-understood and it is thought to participate mainly in the clock output [2]. Both CRY proteins inhibit the transcription of both *cry*s and *per*s by binding to the BCC. A fifth feedback loop models the positive feedback mediated by the REV-ERBα protein, which modulates *cry1* transcription. The effect of REV-ERBα on *clk* expression is omitted in the model. The complex pattern of phosphorylation of PER species by the CK1 family is simplified so as to occur in two stages, performed by one kinase C (as named in the model) at constant concentration, which plays the role of active kinase concentration. A primary phosphorylation allows for binding to CRY and nuclear transport. A secondary phosphorylation, which only occurs for Per1, prohibits nuclear entry. The BCC concentration is modeled as having a constant value, which is a simplification, as *bmal1* is rhythmically expressed, probably under positive feedback control of PER2 [12] and retinoic acid–related orphan receptor (ROR) [22] as well as negative feedback control of REV-ERBα [8]. The model's authors set values for the 38 parameters through a combination of experimental data available from mouse suprachiasmatic nucleus (SCN) and liver cells, and fitting to overall system behavior [18]. Under constant darkness conditions, the model is an autonomous oscillator of the limit cycle type and has a FRP of 24.3 h. The model encapsulates mathematically much of what is known about the mammalian circadian biochemistry, with a few omissions and simplifications. New discoveries will undoubtedly lead to improved versions of the model. As is, it is an excellent basis for theoretical investigation of this interesting and important network control system. The model agrees well with wild-type and mutant behavior.

Circadian clock models are generally limit cycle oscillators, a characteristic of which is robustness of period and amplitude with respect to perturbations in their state variables (corresponding to concentrations or activities here). Mathematically, such systems will asymptotically approach the limit cycle trajectory from any initial condition in the region of attraction of the limit cycle. The shape and situation of the limit cycle depends only on the parameters of the system. This property, however, can make it more difficult to study these systems. Without knowing the exact limit cycle trajectory, iterations over several periods of oscillation are needed to approximate it. It is not clear a priori how many periods are needed to reach the limit cycle to a given tolerance. At the same time, the exact limit cycle properties (period, amplitudes, relative phases) are of direct biological interest. In this article, we present a new method for the exact computation of sensitivity trajectories of limit cycle oscillators and sensitivities of derived quantities with respect to model parameters. Sensitivity analysis probes how a small variation of a parameter or initial condition away from a reference solution influences the trajectories of the state variables, and of derived quantities. Applied to biological systems, sensitivity analysis can help to analyze how changes in rate parameters or temporary perturbations in protein or mRNA concentrations can influence the behavior of a system. It is becoming a standard tool for systems biologists. The use of various sensitivity metrics has been explored in a variety of network biology studies [23,24], including a number of simpler models of circadian rhythms [25–27]. However, the exact sensitivity analysis of oscillating systems is more challenging than for other dynamic systems [27,28]. It has been shown that the parametric sensitivities of periodic systems can be decomposed into a bounded and an unbounded part according to

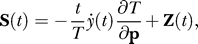
where **Z**(*t*) is the periodic matrix containing the parametric sensitivities at constant period. This part is sometimes referred to as the “cleaned out” sensitivities [29], as opposed to the “raw” sensitivities **S**(*t*), and is reported to contain information on shape and amplitude of the oscillation [30]. In order to achieve this decomposition, two conditions have to be met. First, the exact computation of the period sensitivities 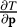 is required. Second, appropriate initial conditions for **S**(0) = **Z** (0) have to be found. Since one is interested in initial conditions of the state variables that lie on the periodic orbit, which in its shape and location depends on the system parameters, those initial conditions are not independent of the parameters. Consequently, the sensitivities cannot be initialized with the zero matrix as is usual in other dynamic systems. An incorrect initialization of the sensitivities leads to an unbounded error of unknown magnitude [28]. In the current work, we have derived a rigorous procedure for computing the sensitivity of the period of a limit cycle oscillator with respect to model parameters and applied this to the most detailed model of circadian rhythms available [18]. This has provided a particularly high-resolution view of the role different model elements play in setting the period of oscillation and for the first time to our knowledge highlighted that reactions involving Per2 have an especially strong effect on the period. Interestingly, the results point to a series of steps forming a reaction cycle, rather than to any particular step in that cycle.

We describe and apply a strategy that identifies the exact limit cycle trajectory by solving a boundary value problem (BVP) and then using the solution of this BVP to calculate the exact sensitivities of state variables, amplitudes, and period of the oscillation without resorting to the iterative methods typically used for limit cycle systems.

Here, we applied our sensitivity analysis methodology to study the limit cycle circadian clock model of Forger and Peskin [18]. Because the same parameters are used in multiple places throughout the model, the individual sensitivities were computed on a per-reaction basis, rather than a per-parameter basis. Due to parameter sharing, the 38 model parameters describe 231 reactions in the model. Sensitivity analysis was performed using both the original 38 lumped parameters or with an unlumped parameter set in which the effect of each of the 231 reaction rate parameters was probed individually. The unlumping as described in Materials and Methods does not change the physical model but rather provides a more detailed analysis of the roles of individual physical and chemical reactions. Meaningful results were only obtained in the unlumped calculation, rather than the lumped sensitivities obtained when analyzing the original model parameters directly, which simultaneously affected multiple reactions. This analysis revealed that the period setting is strongly dominated by processes within the Per2 feedback loop, but not the subtly different Per1 loop. The mechanism of this responsibility distribution is elucidated in several numerical experiments and supported by published experimental results. Moreover, a potential mechanism for short-term period adjustment is identified and discussed, namely the activity of CK1 isoforms.

## Results

### Most High-Impact Parameters Are Located in the Per2 Loop

**Figure 2:**
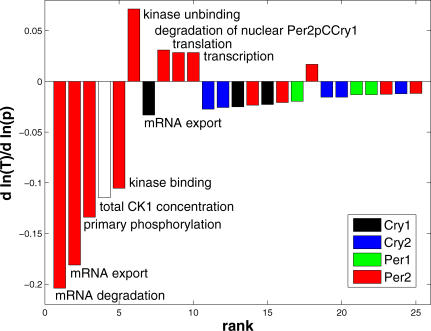
Top 25 Ranked Sensitivities Ordered by Relative Period Sensitivity Black bars indicate Cry1-related parameters, blue bars indicate Cry2-related parameters, green bars indicate Per1-related parameters, and red bars indicate Per2-related parameters. The white bar represents a single, special parameter representing the total kinase concentration. Where Cry- and Per-related parameters overlap, the Per species colors are shown. “Per2pCCry1” describes a complex of once-phosphorylated PER2 with the kinase C and CRY1.

The relative sensitivities of the FRP *T* with respect to each parameter *p_j_* on a per-reaction basis, 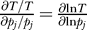 , were calculated, and then rank-ordered by magnitude. Results for the top ten ranked sensitivities are graphically represented in Figures 1 and 2. Per2-related reactions dominate by far in their influence on the period in the system. Eight of the ten highest-magnitude sensitivities are Per2 related (including expression, transport, reaction, and degradation of Per2), and only one is not directly related to the Per2 feedback loop (nuclear export rate of Cry1 mRNA). The overwhelming dominance of Per2 processes in influencing clock period in the model is particularly interesting given that *per2* mutations are linked to FASPS [14].

Figure 2 shows that the top-ranked sensitivities are significantly larger than the remainder, indicating a strongly localized distribution of sensitivity of the period within the network. At the same time, there is no single parameter (and therefore process or reaction) found to be the only control for increasing or decreasing the period of oscillation; rather, the period-setting responsibilities are shared among a number of processes within the Per2 negative feedback loop. Likewise, the localization of high sensitivity within the network cannot be attributed to a class of reaction or process (such as phosphorylation, translation, or transcription).

In order to test the influence of the exact network parameterization on the results shown here, additional parameter sets were created (see Materials and Methods). The period sensitivity rankings of all modified parameter sets correlate highly with the nominal parameter set (Spearman rank correlation factors between 0.890 and 0.966), which is surprising given the large number of parameters with negligible period sensitivity. The majority of the top ten parameters shown in Figure 2 (6.3 of them on average) are found in the top ten of the modified sets, an average of 4.3 of the original top five parameters are found in the top five of the modified sets, and the original top six parameters are represented in nine of the modified top ten. The casein kinase concentration ranks sixth or higher in all but two of them, and tenth or higher in all but one. An average of 5.8 parameters in the top ten is Per2-related. Thus, the results presented here do not depend very strongly on the particular parameter values in the Forger and Peskin model, but instead appear to be a property of this class of models in the neighborhood of the parameterization developed by Forger and Peskin [18].

### Differences Between the Per1 and Per2 Loops

Although Per1 and Per2 carry out similar reactions, only Per2 is singled out as highly significant in affecting the period of oscillation. Model dissection was used to analyze the source of this difference. There are four differences between Per1 and Per2 in the original model. One is a topological difference, in that PER1 can be phosphorylated a second time, which masks its nuclear localization sequence; doubly phosphorylated PER1 or any of its complexes cannot enter the nucleus. The remaining three differences are purely parametrical and of different relative magnitude—differences in transcription rates (the *per1* rate being 2.6-fold higher), rates of first phosphorylation (the PER2 rate being 5-fold higher), and mRNA degradation rates (the Per1 rate being 16-fold higher). These differences cause the PER2 concentration to be roughly 2.5 times that of PER1, with minima and maxima occurring at almost the same times; the PER1 concentration is not negligible, however. The loss of Per1 alone does not abolish rhythmicity, but the loss of Per2 alone leads to a slowly decaying amplitude of the oscillation [18].

Each of the differences was studied in individual numerical experiments. In a first set of numerical “mutations,” the rates that are different between Per1 and Per2 were made equal at either the value of the Per1-specific rate or the value of the Per2-specific rate. Then, the sensitivity analysis was repeated, and the ranking of the resulting sensitivities was compared with the ranking shown in Figure 2 (unpublished data). In short, the findings pointed toward the mRNA degradation rate as well as the rate of primary phosphorylation being influential in making Per2 the period-setting feedback loop.

**Figure 3:**
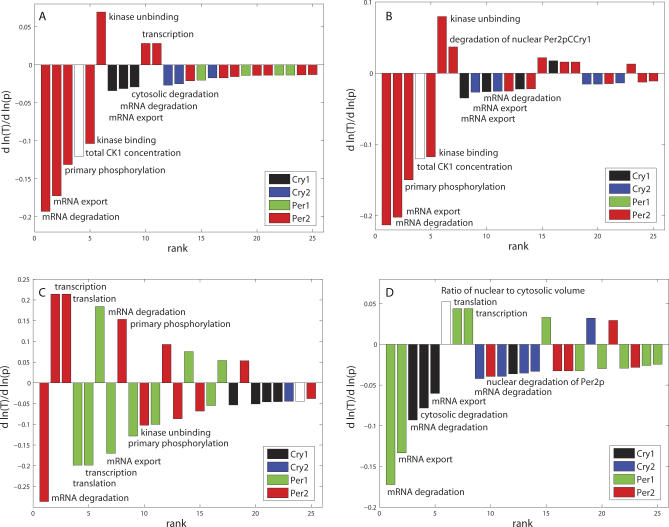
Top 25 Ranked Period Sensitivities for Different Numerical Experiments—Flipped Rate Parameters Flipped rate parameters between Per1and Per2 for (A) secondary phosphorylation rate, (B) transcription rate, (C) primary phosphorylation rate, and (D) mRNA degradation rate. Black, Cry1-related parameter; blue, Cry2-related parameter; green, Per1-related parameter; red, Per2-related parameter. Where Cry and Per related parameters overlap, the Per species colors are shown.

In order to observe this effect more clearly, the rates of the same reactions were reversed in the next set of numerical experiments, before repeating the sensitivity analysis and ranking comparison as before. The results are shown in Figure 3. Neither the only topological difference nor the different transcription rates for *per1* and *per2* are crucial for the differential behavior of the two homologues (Figure 3A and 3B), as the sensitivity ranking remains largely unchanged.

When the rates of primary phosphorylation were reversed, the maximum sensitivity in the network increased. While the highest magnitude sensitivity remained the Per2 mRNA degradation rate, Per1-specific rates now appeared almost alternating with the Per2-specific rates, as if in this scenario the two share the period-setting responsibility (Figure 3C); yet, the overall concentrations of Per1 and Per2 concentration remain largely unchanged.

When the rates of mRNA degradation were reversed for both genes, a subset of Per1-specific rates moved up in the ranking, and at the same time, the maximum sensitivity found decreased in magnitude significantly (Figure 3D). Per1 in this scenario dominated the period-setting, and its concentration was now a factor of ∼20 larger than the Per2 concentration.

**Figure 4:**
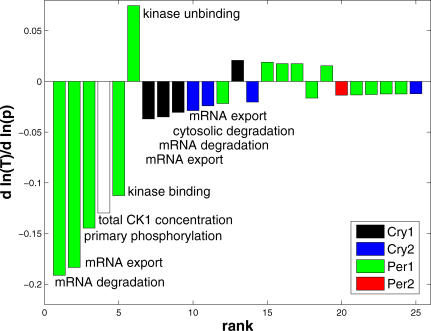
Top 25 Ranked Period Sensitivities When mRNA Degradation Rate and Primary Phosphorylation Rate for Per1 and Per2 Were Reversed The color assignment is identical to Figure 2.

When both of the rates for mRNA degradation and primary phosphorylation were reversed simultaneously, a clear role reversal between Per1 and Per2 occurred (Figure 4). We can thus say that the combined action of mRNA degradation and primary phosphorylation of Per2, in comparison to Per1, are what cause the Per2 loop to dominate in setting the period of the circadian clock.

It should be noted that the rates of mRNA degradation in the original model are more different in a relative sense than the rates of primary phosphorylation. The quantitative results obtained here may vary upon a more exact determination of the parameter values used in the model. However, the qualitative insights gained from the numerical experiments performed in this work appear to be robust to changes in parameter values.

### The CK1 Kinase Activity Alone Can Alter the Period over a Wide Range

**Figure 5:**
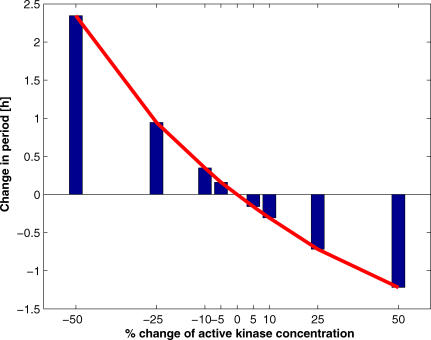
Changes in the Period as a Result of Variation in Kinase CK1 Concentration All other parameters remained constant.

**Figure 6:**
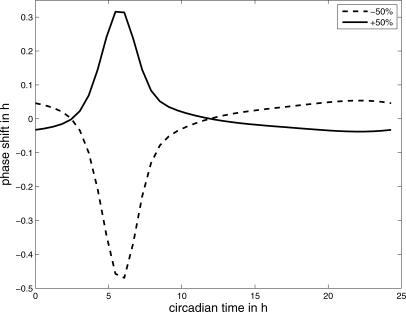
Phase Response Curve for CK1 Concentration The phase response indicated by the ordinate is the permanent effect that was caused by a 50% up or down shift in active kinase concentration of 30 min duration. A positive phase shift indicates a phase advance, a negative one phase delay. The starting time of the step change corresponds to the time indicated by the abscissa. Circadian time zero corresponds to dawn.

The analysis of period sensitivities identified the total active concentration of CK1 isoforms as well as the kinase-binding kinetics as being among the main determinants of the period. As the sensitivity analysis measures the effects of local (infinitesimal) parameter variations, a possible mechanism for modulating the period of the system through modulating the amount of active kinase in the system was verified by parametric studies. The results are shown in Figure 5. The kinase concentration allows for modulation of the period over a wide range of parameter values. By varying the parameter 50% up or down, the period was changed by −5% or +9.7% (−1.25 to 2.4 h), respectively. The sensitivity of the period with respect to kinase concentration remains negative, as the period becomes shorter with increasing kinase concentration and vice versa, over the entire range. It is assumed that a temporary change in the period will cause a permanent phase shift, a process called “parametric entrainment” in the circadian literature [2]. The results shown in Figure 5 suggest that by modulation of the kinase concentration, it is relatively easier to produce a phase delay (a temporary decrease of kinase concentration, leading to a temporarily longer period) than it is to produce a phase advance, as shown by the larger magnitude period variation achieved for a 50% decrease in kinase concentration compared with a 50% increase. A similar difference is noted in the maximum amplitude of the phase response curve (PRC) shown in Figure 6, where the phase shift as the result of a short-term step change in active kinase concentration is shown as a function of time. This figure shows, again, that the same absolute change in kinase concentration at the right time results in a longer delay but shorter advance. The results also show that the magnitude of phase shift depends dramatically on when during the day the kinase modification is applied, both for delay and advance. It should be noted that changes in period shown in Figure 5 correspond to a stationary property of the system. In contrast, the response measured in Figure 6 describes a transient effect of a short-term disturbance of the system—the short-term parameter change does not allow for the system to approach its perturbed stationary state.

## Discussion

### The Per2 Loop Sets the Period

The results presented for the circadian clock system in mammals suggest a strongly localized distribution of functionality within the network. Through detailed analysis of the period sensitivities, it was shown that the period of oscillation is set by reactions distributed throughout the Per2 negative feedback loop. The ten parameters with the greatest period sensitivities are dominated by Per2-related species; likewise, Per2-related reactions have high sensitivities.

Within the Per2 feedback loop there is a self-consistency as to the effects on the length of the period; changes that accelerate (decelerate) progress through the loop lead to a shorter (longer) period. For example, multiple processes that reduce the half-life of Per2 mRNA all produce a shortened period. These processes include faster mRNA degradation, faster mRNA export, and interestingly, *slower* transcription. Likewise, faster kinase binding or phosphorylation lead to faster migration of CRY1-bound species into the nucleus, which closes the negative feedback loop faster and results in a shorter period. Interestingly, PER2 and CRY1 interoperate to control the rate of nuclear transport of the phosphorylated complex. Changes that prolong the half-life for CRY1 in the nucleus (faster dissociation of PER2-bound CRY1 species and slower CRY1 degradation) result in a longer period, a fact that was recently confirmed in experimental studies [31]. Changes can also be understood through their effects in altering concentrations. Increases to the cytosolic concentrations of CRY1 (faster nuclear export of CRY1 and bound species, faster transcription, or faster translation) lead to shorter periods. Because unbound CRY cannot migrate from nucleus to cytosol, a delay of CRY1 in the nucleus slows the feedback from the Per2 loop and lengthens the period. Processes that increase the amount of Per2 produced (faster Per2 transcription or translation, or slower mRNA degradation) tend to lead to a longer period. This self-consistency makes intuitive sense of the network structure–function relationship computed here and lends additional support to the notion that the results are not overly dependent on the details of the particular model implementation here.

Published experimental results indicate that the roles of Per1 and Per2 in the circadian clock mechanism are not redundant [13,16]. Our findings confirm these results on a network analysis level: the Per2 feedback loop carries responsibility that Per1 does not share. Due to the detailed and comprehensive model, the mechanistic detail behind this organization could be analyzed to show that the values of two reaction rates, the rates of mRNA degradation and of primary phosphorylation of Per2, cause this feedback loop to dominate period-setting. In another computational study [32], mRNA degradation rates were found to influence strongly the period of oscillation throughout a set of four different circadian clock models, not including the model studied here. The present study provides further insight in that it is mainly only one mRNA degradation rate, that of Per2, that matters most. Even the second most sensitive mRNA degradation rate, that of Cry1, is only 20% as significant with respect to the period.

It should be noted that the authors of the model also report a sensitivity called “sensitivity of the badness of the fit” [18]. This quantity is only indirectly related to the period sensitivity, and is also only computed for the 38 “lumped” original parameters. Without dissecting the multiple roles of the original parameters, the Per2 loop cannot be identified as the period-setting feedback loop, nor does it become obvious how the period-setting responsibility is distributed throughout the loop.

### The Positive Feedback Loop Might Not Participate in Period-Setting

The sensitivities of the period with respect to all parameters associated with the Rev-Erbα loop were zero, suggesting that the Rev-Erbα loop may not participate in setting the period for this model. This hypothesis was confirmed by removal of the entire loop without consequence for the period (unpublished data). Interestingly, this is an area where the model appears to disagree with experiment; experiments show that while Rev-Erbα is not essential for rhythmicity, period length and phase-shifting behavior are altered in null mutants [8], although less so than in Per2 null mutants [15,16].

In this portion of the model, the action of REV-ERBα on Bmal1 expression is omitted, and the Rev-Erbα loop is parameterized in such a way that the resulting concentrations are essentially zero [33]. To test whether inaccuracies in the Rev-Erbα portion of the model could compromise conclusions regarding the role of the Per2 loop, simulations and sensitivities were computed for artificially manipulated versions of the model that substantially increased the activity of the Rev-Erbα loop. Even when the flux through the Rev-Erbα loop was increased by five orders of magnitude (by increasing the transcription and translation rates and decreasing the degradation rates), and the corresponding sensitivities became significant and moved up the ranks (ranging from 168th to 225th in the original parameterization versus 68th to 168th for the increased flux model), the top 50 ranking parameter sensitivities did not change significantly.

Furthermore, in accordance with recent findings in the experimental literature [22,34], we have constructed an alternate version of the model (Dataset S3) with a sixth feedback loop involving ROR. This receptor was found to have an opposing role to Rev-Erbα in the control of Bmal1 expression. While Bmal1 was still not explicitly represented, we included the indirect effect of ROR as well as that of Rev-Erbα on the transcriptional activity of all circadian genes in the model. This change resulted in the use of four new state variables and ten new parameters. The preliminary parameterization of this sixth feedback loop was done using qualitative insights from the experimental data, and chosen so that the peak in ROR would follow the peak of REV-ERBα in the nucleus and so that transcriptional control of ROR would be similar to that of Per2. The transcription rate of *rev-erb*α was increased 100-fold to make the corresponding concentrations more significant. Those modifications to the original model did not alter mutant behavior, period, or the results presented in this paper. The ranking of relative period sensitivities remains virtually unchanged. The ten new parameters ranked between 148 and 218 out of 241. This suggests that while there are still discrepancies between the known biology and the model that will undoubtedly be resolved through future work, the results shown here are not sensitive to changes in this part of the model.

### A Potential Mechanism for Accelerating or Decelerating the Oscillation

It is of interest for the understanding of network design, as well as for potential therapeutic strategies, to identify possible points of intervention for period control. Furthermore, it is known that parametric entrainment (which relies on period modulation to control the phase of oscillation) plays a role in the mammalian circadian clock, although details are not understood [2]. The total CK1 concentration appeared fourth in the rank-ordered sensitivities, and is a quantity that deserves special attention. While it is modeled here as a constant quantity, it realistically represents a concentration of active kinase, which may not be constant throughout the cycle. Some of the other top ten parameters can be modified by genetic mutation on a long timescale (such as the mRNA degradation rate), or a medium timescale (such as transcriptional regulation). However, the (active) kinase concentration could potentially be regulated both on a very short timescale by post-translational modification through an input signaling pathway or a medium timescale through transcriptional regulation. In fact, CK1ɛ is known to inactivate itself by autophosphorylation, a process that is counteracted by cellular phosphatases [35]. It has been suggested that such phosphatases can be activated by signaling pathways such as the Wnt pathway [36] so as to activate CK1ɛ as a result of a signaling cascade.

A comparison with experimental results for period-related abnormalities reveals that PER2 phosphorylation by CK1 has significant involvement in setting the period. It has been shown experimentally that in individuals with one type of FASPS, the human *per2* gene is mutated at the site of its phosphorylation by CK1ɛ. This mutation causes hypophosphorylation and ultimately a phase advance, which is typically associated with a shortened FRP [14]. In individuals with another type of FASPS, the *ck1δ* gene is altered [37].

In hamsters, a mutation called *tau* in *ck1ɛ* causes a short circadian period. For the Forger and Peskin model, an increased rate of primary Per2 phosphorylation predicts a shortened period. This finding contradicted the prior observation that the *tau* mutation was a loss-of-function mutation of CK1ɛ in in vitro experiments [38], thus leading to the discovery of the differential action of CK1ɛ on clock-related versus generic substrates. Recent findings have confirmed that the *tau* mutation is in fact a gain-of-function mutation with respect to the phosphorylation of PER2 [39].

It should be noted that the exact pattern and functional consequences of Per2 phosphorylation are simplified in the model [18]. More recently, its details have been investigated [9], providing additional insight. In broad terms, there are two effects of PER2 phosphorylation. The phosphorylation site involved in FASPS (Ser 659) was shown to increase nuclear retention and stabilization of PER2. Phosphorylation at other sites of PER2 leads to increased degradation. Only the latter effect is represented in the model used for this. In order to substantiate the results in this study in the light of more recent experimental data, the phosphorylation pattern suggested in [9] was incorporated into the model. No new species were created, because the original model already includes a doubly phosphorylated PER2 species. Only three rate parameters were added to the model (nuclear import rate of PER2pp species, and modified degradation rates for nuclear and cytosolic PER2pp species). The previously unused secondary phosphorylation rate of PER2 was reassigned. Parameter values were chosen to closely reflect the relative rates as published in [9], based on the rate values in the original model; i.e., the rate of secondary phosphorylation was set equal to that of primary phosphorylation, as suggested in [9], at the value published in [18]. The modified model in MATLAB format can be found in Dataset S2. This very preliminary parameterization resulted in a period of 23.82 h, and the mutant behavior with respect to knockouts as described in [18] was unchanged. Again, the parameters were unlumped, and the sensitivity analysis and ranking was repeated. The top six parameters are the same in sign and very similar in magnitude as those of the original model, confirming the dominant role of Per2 again. The twice-phosphorylated PER2 species appears twice in the top ten (ranks seven and nine, unbinding from CRY1 and degradation, respectively), and the unbinding and binding kinetics between CRY1 and CLK:BMAL1 take ranks eight and ten, respectively. The period sensitivity with respect to the secondary phosphorylation rate is smaller than that of the primary (rank 64 versus rank three), and positive, as expected. Thus, the inclusion of more recently discovered mechanistic detail regarding phosphorylation confirms the result that phosphorylation of PER2 and Per2-related nuclear trafficking are key period-setting reactions.

It was previously shown in experiments that the circadian clock in humans, as well as in mice, takes longer to phase advance than phase delay, if exposed to jet lag conditions in the form of a 6 h time shift during daylight hours [6]. Jet lag is a transient phenomenon that in this case lasted several days, during which the organism is thrown off the steady-state periodic cycle and resets its new phase according to an entraining signal. While the PRC provides some insight in the phase-shifting behavior of the clock in response to an outside stimulus, relating this steady-state response to a transient, jet lag situation is generally difficult. During jet lag, the relative timing between “clock time” and “entrainment time” changes continuously, the system is not at its steady state, and the response to a stimulus in a (nonlinear) limit cycle system depends on the state of the system at which the stimulus is received. In fact, a recent computational study shows that designing an optimal input stimulus for rapid phase resetting is nontrivial even if the PRC is well known [40].

In the following discussion, the focus is therefore not on the exact mechanism of overcoming jet lag in the mammalian circadian clock model, but rather the apparent similarities in the asymmetry between phase delay and phase advance between observations in jet-lagged mice and the numerical experiments performed in this work. It is sometimes argued that this difference is caused by the FRP being longer than 24 h; however, the results presented here are in reference to the innate FRP of 24.3 h. New experimental results have furthermore identified the FRP in humans to be closer to 24 h than previously reported [41,42].

It is apparent that there could be a number of places in the network through which phase shifts can be introduced. For example, it has been suggested that Per1 is important during discrete entrainment, the phase response to transitions in the light stimuli, and is especially receptive to such signals during the night [43]. During the 6 h advance phase shift induced in the experiment by Reddy et al., the light onset occurred in the middle of the former night, temporarily inducing Per1 mRNA [6]; however, the phase shift achieved in this experiment was markedly slower than the phase delay response. To produce the phase delay, the new light onset corresponds to the former noontime, which coincides with the time at which the kinase concentration is most influential as seen in Figure 6. While the discussion here is solely circumstantial, and we have no formal proof that the kinase concentration is involved in the asymmetric phase shifting reported in [6], our observations in Figures 5 and 6 show similarity in the sense that phase delay is accomplished relatively more easily than phase advance. In addition, the particular shape of the PRC in Figure 6 shows a large bias toward phase advance (delay) for increased (decreased) kinase activity; in other words, the PRC itself is asymmetric. If the kinase concentration is modulated throughout the cycle, the PRC suggests an effect only in the desired direction or else, if the modulation happens at a phase-shifted time, little effect at all. This could make the adaptation to a new phase during a transient situation such as jet lag easier to control. Thus, we hypothesize that the kinase concentration (activity) could be a particularly convenient control point. It may be used by the natural system, for example, as an additional way to process entrainment inputs during the day, especially those of long signal duration, thus acting as the control element for continuous entrainment discussed earlier. The kinase concentration may also be useful as a therapeutic point of intervention. Figure 5 shows that a 50% change in kinase concentration can lead to a 1 h to 2 h phase shift per day, and larger changes increase that shift further.

While the exact molecular biology of the phase advance versus delay response is beyond the scope of a purely computational study, it is discussed next that a molecular basis for differences between phase advance and phase delay can be identified for this model system. The mechanistic reason for the differences between phase advance and delay in this model is that once PER2 is phosphorylated, two processes compete for it. Phosphorylated PER2 can be either degraded or bound by the CRY proteins, which protects them from degradation. Increased kinase activity results in more phosphorylated PER2 being formed, but it is also degraded at a higher rate. Therefore, the feedback loop is accelerated. In comparison, if the kinase activity is decreased, the PER2 concentration in the cytosol increases until the rate of phosphorylation, which is proportional to the product of PER2 concentration and kinase concentration, is equal to the maximum phosphorylation rate in the wild type. No process is competing with the slowed-down phosphorylation rate and the phase-delayed nuclear import of the PER–CRY complex.

Taken together, these results suggest that control of the active kinase concentration is a possible way for the system to modify the period (therapeutically or naturally), especially on short timescales and following entrainment signals received during daytime.

This study illustrates computational approaches for probing structure–function relationships in network models—namely by showing how sensitivity analysis of a sufficiently detailed mechanistic model can relate theoretical results to experimental findings. The technique can be used both for refining the biological model and understanding the implications of network design for normal operation, disease, and therapeutic intervention.

## Materials and Methods

### The boundary value problem.

An ODE model for a biological system is analyzed, where 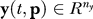 are usually concentrations of protein, mRNA, or other species. The parameters 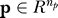 are typically reaction rate constants in mechanistic models, or lumped rates of processes such as transport between compartments.

Given a fixed value for **p**, initial conditions on the limit cycle and the period of the oscillator are identified by solving a BVP for initial condition **y**_0_(**p**) and period *T*(**p**) subject to a periodicity condition

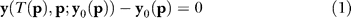
and a phase-locking condition

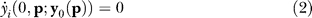
for some arbitrary 
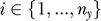
 , with the limit cycle trajectory **y**(*t*, **p**; **y**_0_(**p**)) given by the solution of

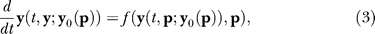
and **y**(0, **p**; **y**_0_(**p**)) = **y**_0_(**p**). From this, we obtain initial conditions for the state variables that lie on the limit cycle. The (*n_y_* + 1)*^st^* condition in Equation 2 fixes the solution to a point on the limit cycle where the state variable *y_i_* is stationary. Any arbitrary state variable can be chosen for this constraint.

This BVP was solved using NITSOL, an inexact Newton solver [44], and CVODES, a stiff ODE solver with sensitivity analysis capabilities, for the integration of the dynamic system [45].

### Sensitivity analysis for limit-cycle oscillators.

In most dynamic systems, the parametric sensitivities 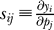 for a system such as Equation 3 are integrated from zero initial conditions according to

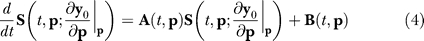
where 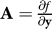 and 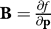 .

In the case of the solution of a BVP in a limit cycle system, Equation 4 still applies; however, setting the initial conditions to zero would not be correct. The initial conditions **y**_0_(**p**) are now dependent on the parameters. Because this dependency is implicit through the solution of Equations 1 and 2, it is not immediately clear how to set the initial conditions 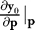 for the system in Equation 4 correctly. This problem is solved as follows.

The set of Equations 1 and 2 can be differentiated with respect to the parameters **p**, and written in matrix form yielding the following expression

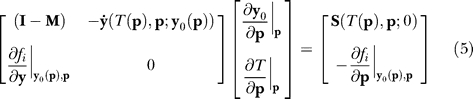
where** I** is the *n_y_* × *n_y_* identity matrix, and **S**(*T*,**p**;**0**) is the solution at time *T*(**p**) of sensitivity Equation 4 for zero as the initial condition. The matrix **M** is 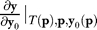 , the matrix of sensitivities of the state variables with respect to their initial conditions at *T*(**p**). This matrix is also called the Monodromy matrix of the sensitivity system. For more detailed explanation, see [28].

The matrix Equation 5 can be solved for the matrix of unknowns. The calculation of the sensitivity trajectories 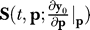 can then easily and exactly be performed by integrating Equation 4 starting from 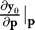 , allowing the decomposition into a periodic part and an unbounded part as described previously. This method, in contrast to some previous publications [27,46], enables the exact computation of the period sensitivities rather than approximating the result by truncation of a limit or by integration of the entire system for a sufficiently long time, resulting in significantly less computational effort and, in principle, exact results.

All matrix manipulations were performed in MATLAB 7.4.0 (R2007a).

The circadian clock model was obtained as a MATHEMATICA file from the supplementary materials of [18] at http://www.pnas.org/cgi/content/full/2036281100/DC1/6 and was rewritten as MATLAB code (available in Dataset S1).

### Unlumping of the parameters.

The original model has 38 rate parameters, many of which are used in multiple roles within the reaction network; e.g., the mRNA export rate is the same for all mRNA species. In a “lumped” sensitivity analysis, a parameter may be shown to have great impact on the period of oscillation. However, on a network analysis level, one is interested to see which of its multiple roles is the most important in the setting of the period. Therefore, the parameter was “unlumped,” meaning new parameters were assigned to each species that is affected by it so that each new parameter corresponds to a unique chemical reaction or physical process. The parameter values of all those were the same as the value of the original, single parameter. In other words, the model itself did not change during this process, but it is now possible to distinguish the different roles a parameter might play. Doing so does not necessarily imply that the organism has the capability to independently control the unlumped parameters.

### Alternative parameter sets.

The original set of 38 parameters was modified by first randomly choosing ten parameters, then by randomly modifying their value either by a factor two up or down. The resulting model was simulated over 40 nominal periods in order to approach the limit cycle. If the apparent period (time difference between the last and second-to-last minimum in the CRY1 concentration) was between 23.5 h and 25 h, the model was subjected to the BVP solver. This selection criterion was chosen because it is known that the mammalian clock oscillates roughly in this range of periods, and it is irrelevant to investigate parameterizations with known, unphysical periods. A total of 15 such models were generated, and 11 out of those were converged easily; the others were discarded. Possible reasons for nonconvergence include the presence of damped oscillations, which would not have been detected in the earlier test. By inspection, it was found that the modified parameters included both low- and high-sensitivity parameters in the nominal model. The parameter values of the 11 final models are found in Table S1, along with the resulting period and ranking of the top 25 sensitivities.

## Supporting Information

Dataset S1 MATLAB Model of the Mammalian Circadian Clock as Published by Forger and Peskin (16 KB DOC)

Dataset S2 MATLAB Model of the Mammalian Circadian Clock with Phosphorylation Details Modeled after Findings by Vanselow et al. (15 KB DOC)

Dataset S3 MATLAB Model of the Mammalian Circadian Clock with ROR Feedback Loop Added (16 KB DOC)

Table S1 Alternate Lumped Parameter Sets, the Resulting Period of Oscillation, and Rankings of the Corresponding Top 25 Scaled, Unlumped Sensitivities (97 KB PDF)

## Abbreviations

BCC: BMAL1–CLK complex
BVP: boundary value problem
CK1: casein kinase 1
FASPS: familial advanced sleep phase syndrome
FRP: free running period
ODE: ordinary differential equation
PRC: phase response curve
ROR: retinoic acid–related orphan receptor
