# Supplementary material for: The Per2 Negative Feedback Loop Sets the Period in the Mammalian Circadian Clock Mechanism
Source: PLoS Comput Biol. 2007 Dec 14;3(12):e242. doi: 10.1371/journal.pcbi.0030242 (PMC2134962; doi:10.1371/journal.pcbi.0030242)
Supplement: Supplementary file 1 [file pcbi.0030242.st001.pdf]

Supplementary materials to Wilkins, Barton & Tidor (PLoS Computational Biology).

Supplementary Table 1: Alternate lumped parameter sets, the resulting period of oscillation and rankings of the corresponding top 25 scaled, umlumped sensitivities $\frac{\partial \ln T}{\partial \ln p_j}$. Boldface corresponds to modified parameter values. Nominal lumped rank is the ranking of period sensitivities when the parameters were not unlumped and period sensitivity analysis was performed on the original 38 parameters as published by Forger and Peskin. Orange – Nominal, lumped rank 1-5, Yellow – Nominal, lumped rank 6-10, Blue – Per2 related rate parameter, Pink – Kinase concentration.

j	**1**	**2**	**3**	**4**	**5**	**6**	**7**	**8**	**9**	**10**	**11**
**1**	10.0	**20.0**	10.0	10.0	10.0	10.0	10.0	10.0	10.0	10.0	**20.0**
**2**	1.031	1.031	1.031	1.031	1.031	1.031	1.031	1.031	1.031	1.031	1.031
**3**	2.533	2.533	2.533	2.533	2.533	2.533	2.533	2.533	**5.067**	2.533	**1.267**
**4**	**6.784**	3.392	**1.696**	**6.784**	3.392	**1.696**	**1.696**	**1.696**	3.392	3.392	**1.696**
**5**	**0.165**	0.082	**0.165**	0.082	0.082	**0.165**	**0.165**	0.082	**0.165**	0.082	0.082
**6**	0.090	0.090	**0.045**	0.090	0.090	**0.045**	0.090	**0.180**	0.090	**0.180**	**0.180**
**7**	0.436	**0.872**	0.436	**0.872**	0.436	0.436	0.436	0.436	0.436	**0.872**	0.436
**8**	0.594	0.594	0.594	**0.297**	0.594	0.594	**1.188**	**0.297**	**0.297**	0.594	0.594
**9**	0.465	0.465	0.465	0.465	0.465	0.465	0.465	0.465	0.465	0.465	0.465
**10**	5.087	**10.173**	5.087	5.087	**10.173**	5.087	5.087	5.087	**10.173**	5.087	5.087
**11**	0.266	0.266	**0.133**	0.266	0.266	**0.133**	0.266	**0.533**	**0.133**	0.266	0.266
**12**	0.352	0.352	0.352	0.352	**0.176**	0.352	0.352	0.352	0.352	0.352	0.352
**13**	2.307	**1.153**	**1.153**	2.307	2.307	**1.153**	**1.153**	**1.153**	2.307	2.307	2.307
**14**	0.713	0.713	0.713	0.713	0.713	0.713	0.713	0.713	0.713	**0.357**	0.713
**15**	0.836	**0.418**	0.836	0.836	0.836	0.836	0.836	**0.418**	0.836	**1.673**	0.836
**16**	**0.143**	**0.143**	**0.143**	0.286	0.286	**0.143**	0.286	0.286	**0.572**	0.286	**0.572**
**17**	1.453	1.453	1.453	**0.727**	1.453	1.453	1.453	1.453	**0.727**	1.453	1.453
**18**	**0.000**	0.000	0.000	0.000	0.000	0.000	0.000	**0.000**	0.000	0.000	**0.000**
**19**	**15.423**	7.712	7.712	7.712	7.712	7.712	7.712	7.712	7.712	**15.423**	7.712
**20**	**738.3**	1476.5	1476.5	1476.5	1476.5	1476.5	1476.5	1476.5	1476.5	1476.5	**2953.0**
**21**	23.78	23.78	23.78	**47.56**	**11.89**	23.78	**11.89**	23.78	23.78	23.78	23.78
**22**	807.4	807.4	**1614.8**	807.4	807.4	**1614.8**	807.4	807.4	**1614.8**	807.4	807.4

**23**	308.8	308.8	308.8	308.8	308.8	308.8	308.8	308.8	308.8	**154.4**	308.8
**24**	9.034	**4.517**	9.034	9.034	**18.069**	9.034	9.034	9.034	9.034	9.034	9.034
**25**	**15.323**	7.662	7.662	**15.323**	7.662	7.662	7.662	7.662	7.662	7.662	7.662
**26**	0.046	0.046	**0.023**	0.046	0.046	**0.023**	0.046	0.046	0.046	0.046	0.046
**27**	0.420	0.420	0.420	0.420	0.420	0.420	0.420	0.420	0.420	0.420	0.420
**28**	0.210	0.210	0.210	0.210	**0.421**	0.210	0.210	**0.105**	0.210	**0.105**	0.210
**29**	**1.809**	3.618	3.618	**1.809**	3.618	3.618	**7.236**	3.618	3.618	**1.809**	3.618
**30**	0.133	**0.066**	0.133	0.133	**0.066**	0.133	**0.066**	0.133	**0.266**	0.133	**0.066**
**31**	21.76	21.76	**10.88**	**43.53**	21.76	**10.88**	21.76	21.76	21.76	21.76	21.76
**32**	16.25	16.25	16.25	16.25	**32.51**	16.25	16.25	16.25	16.25	16.25	16.25
**33**	6.211	**12.422**	6.211	6.211	6.211	6.211	**3.106**	6.211	**3.106**	**3.106**	6.211
**34**	**0.188**	0.377	0.377	0.377	0.377	0.377	0.377	0.377	0.377	0.377	0.377
**35**	0.299	**0.597**	0.299	**0.597**	**0.597**	0.299	0.299	**0.597**	0.299	0.299	0.299
**36**	15.11	15.11	**7.56**	15.11	**7.56**	**7.56**	15.11	**30.22**	15.11	15.11	**7.56**
**37**	**0.00017**	0.00034	0.00034	0.00034	0.00034	0.00034	**0.00017**	0.00034	0.00034	**0.00017**	0.00034
**38**	115.8	115.8	115.8	**57.9**	**57.9**	115.8	**231.5**	115.8	115.8	115.8	**231.5**

**Period**<br>**dlnT/dlnp rank**	23.96<br>**parameter number**	23.991<br>**parameter number**	24.753<br>**parameter number**	24.849<br>**parameter number**	24.253<br>**parameter number**	24.653<br>**parameter number**	23.96<br>**parameter number**	23.666<br>**parameter number**	24.063<br>**parameter number**	24.413<br>**parameter number**	24.319<br>**parameter number**
**1**	224	34	34	34	34	34	34	34	34	34	34
**2**	17	224	224	224	224	224	224	224	223	224	224
**3**	34	222	17	8	17	17	17	17	16	180	19
**4**	66	17	19	17	19	19	19	19	39	211	17
**5**	19	19	66	231	66	66	66	66	23	17	66
**6**	65	231	91	19	91	91	91	91	9	66	91
**7**	222	66	27	222	64	27	27	21	224	209	223
**8**	91	8	35	66	27	35	223	20	22	24	16
**9**	35	149	56	211	7	56	35	27	1	2	23
**10**	231	91	7	91	35	7	209	38	19	25	39
**11**	7	211	209	65	56	222	7	222	10	40	171
**12**	209	201	222	39	23	40	199	223	56	99	21
**13**	211	27	65	23	39	22	56	201	64	173	1

**14**	27	43	23	56	222	23	171	56	33	93	22
**15**	38	38	39	149	184	39	23	211	27	39	20
**16**	49	56	171	24	163	147	39	165	43	23	27
**17**	39	65	199	2	65	65	16	2	5	190	9
**18**	23	147	223	223	223	231	163	24	41	91	163
**19**	134	23	43	157	38	149	40	16	231	179	33
**20**	180	39	147	40	209	155	25	39	222	97	64
**21**	8	64	231	25	43	58	21	23	8	7	38
**22**	41	157	40	22	25	163	20	173	38	186	222
**23**	157	49	25	1	40	171	64	199	91	223	10
**24**	56	20	211	41	1	49	222	65	122	21	56
**25**	171	51	149	16	22	41	1	209	17	20	24
