# Supplementary material for: The Per2 Negative Feedback Loop Sets the Period in the Mammalian Circadian Clock Mechanism
Source: PLoS Comput Biol. 2007 Dec 14;3(12):e242. doi: 10.1371/journal.pcbi.0030242 (PMC2134962; doi:10.1371/journal.pcbi.0030242)
Supplement: Supplementary file 4 [file pcbi.0030242.sd003.doc]

function Forger_Peskin_model

%Based on the Forger and Pesking model as published in PNAS in 2003. Notation is taken

%from the supplemental material of this publication, and if discrepancies

%were discovered, from the Mathematica version of the model.

%This extended version included a mechanism of Bmal1 transcriptional control (indirect) through

%Rev-Erba and ROR.

%Increased Rev-Erb level 10 fold (trRv) compared to the original.

%parameters from mathematica file - slightly different from Table one in PNAS!!!

k = [10.000007507426437; 1.0306113804294779; 2.533240595047462; 3.392174131760531; 0.08235038322223653; 0.09010086823641558; 0.43586075263171675; 0.5939301343988281; ...

%	tlp	tlr	tlrv	up	upu	hto	uro	urt

%	1	2	3	4	5	6	7	8

0.4650682485639215; 5.086549513975453; 0.26637517504990715; 0.35208765240076423; 2.3068625846024933; 0.7134369508630467; 0.8364310152509935; 0.28622504147320627; 1.4532467336558366; 0; ...

%	ac	dc	ar	dr	nl	ne	ho	hoo	hot	ht

%	9	10	11	12	13	14	15	16	17	18

7.711599056047925; 1476.5235122841377; 23.77792431606627; 807.3899825274443; 308.75858766813644; 9.034446234019176; 7.661529362606101; 4.596363025142135; 0.4203923725237006; ...

%	Ct	bin	unbin	trPo	trPt	trRo	trRt	trRv	tmc

%	19	20	21	22	23	24	25	26	27

0.21037340452340514; 3.617835946402381; 0.13285836190972028; 21.7634595649439; 16.25382347888401; 6.210956900459175; 0.37665288989017; 0.298706036897769; 15.110143877673188; ...

%	arv	drv	binRv	unbinRv	uRv	umPo	umPt	umR	umRv

%	28	29	30	31	32	33	34	35	36

0.0; 115.7612458072891; 50; 0.42; 0.1511; 10; ...

%	Lon	Nf	trROR	tmcROR	umROR	tlROR

%	37	38	39	40	41	42

2.3; 0.71; 16.25; 21.76; 1.0; 1.0];

%	nlROR	neROR	nROR	unROR	binROR-G binRvRv-G

%	43	44	45	46	47	48

%Unlumping of the pxrxmeters

for i=1:241

if (i<=38)

k_unlumped(i) = k(i);

end

if (i>40 && i<=55)

k_unlumped(i) = k(4);

end

if (i>55 && i<=58)

k_unlumped(i) = k(5);

end

if (i>58 && i<=63)

k_unlumped(i) = k(6);	end

if (i>65 && i<=90)

k_unlumped(i) = k(9);	end

if (i>90 && i<=115)

k_unlumped(i) = k(10);	end

if (i>115 && i<=146)

k_unlumped(i) = k(11);	end

if (i>146 && i<=177)

k_unlumped(i) = k(12);	end

if (i>177 && i<=190)

k_unlumped(i) = k(13);	end

if (i>190 && i<=215)

k_unlumped(i) = k(14);	end

if (i>216 && i<=221)

k_unlumped(i) = k(18);	end

if (i>221 && i<=225)

k_unlumped(i) = k(27);	end

if (i>227 && i<=230)

k_unlumped(i) = k(32);	end

if (i>231 && i<=241)

k_unlumped(i)= k(i-193); end

end

k_unlumped(39) = k(1);

k_unlumped(40) = k(2);

k_unlumped(64) = k(7);

k_unlumped(65) = k(8);

k_unlumped(216) = k(16);

k_unlumped(226) = k(28);

k_unlumped(227) = k(29);

k_unlumped(231) = k(35);

%Initixl condition as found by solution of the boundary value problem with the above parameterization

IC = [0.952827000000000	0.000000000000179	1.851210000000000	3.400550000000000	1.569900000000000	2.883790000000000	0.037273900000000	0.002703450000000...

0.014254200000000	0.032783800000000	0.011484300000000	0.000327715000000	0.000045904700000	0.000006382730000	0.000000000012301	0.000000000028278...

0.159858000000000	0.449865000000000	0.094019800000000	0.216675000000000	0.002354010000000	0.027731700000000	0.001166300000000	0.013695900000000...

0.000104148000000	0.000000000000000	0.003222110000000	0.037673000000000	0.002033070000000	0.023724300000000	0.001457580000000	0.000904788000000...

0.000000000000000	0.000000000000000	0.000135623000000	0.000000000000000	0.002778410000000	0.032826100000000	0.001750770000000	0.020644000000000...

0.000977952000000	0.000000000000000	0.000607071000000	0.000000000000000	7.887110000000000	5.047910000000000	0.001921570000000	0.023375500000000...

0.000676521000000	0.008122180000000	0.000078912200000	0.000000000000000	0.003403330000000	0.040110700000000	0.002112300000000	0.024817500000000...

0.000176799000000	0.000000000000000	0.000107952000000	0.000000000000000	0.000023419100000	0.000000000000000	0.009594130000000	0.113683000000000...

0.005946970000000	0.070242100000000	0.000579646000000	0.000000000000000	0.000354277000000	0.000000000000000	0.034736000000000	0.019558300000000...

0.288392000000000	0.002312780000000	0.011177700000000	0.006070000000000	0.000623011000000];

T = 24.2977;

[t,x]=ode23s(@diff_eq,[0 T],IC,[],k_unlumped);

figure;

plot(t,x(:,45));

return

function dxdt=diff_eq(t,x,k)

C = k(19) - (x(19) + x(20) + x(21)+ x(22) + x(25) + x(26) + x(37) + x(38) + x(39) + x(40) + x(41) + x(42) + x(43) + x(44) + x(47) + x(48) + x(51) + x(52) + x(63) + x(64) + x(65) + x(66) + x(67) + x(68) + x(69) + x(70) + x(73));

Rn = x(63) + x(64) + x(65) + x(66) + x(67) + x(68) + x(69) + x(70) + x(71) + x(72) + x(53) + x(54) + x(55) + x(56) + x(57) + x(58) + x(59) + x(60);

%The annotation of species follows the original publication's notation

%exactly

%G

dxdt(1) = k(20)*Rn*(1-x(1)) - k(21)*x(1) - k(240)*x(77) + k(241)*x(16);

%GRv

dxdt(2) = k(30)*x(16)*(1-x(2)) - k(31)*x(2);

%MnRo

dxdt(3) = k(24)*(1-x(1))*(1-x(2))^3 - k(27)*x(3);

%McRo

dxdt(4) = k(27)*x(3) - k(35)*x(4);

%MnRt

dxdt(5) = k(25)*(1-x(1)) - k(222)*x(5);

%McRt

dxdt(6) = k(222)*x(5) - k(231)*x(6);

%MnPo

dxdt(7) = k(22)*(1-x(1))^5 + k(37) - k(223)*x(7);

%McPo

dxdt(8) = k(223)*x(7) - k(33)*x(8);

%MnPt

dxdt(9) = k(23)*(1-x(1))^5 + k(37) - k(224)*x(9);

%McPt

dxdt(10)= k(224)*x(9) - k(34)*x(10);

%MnRv

dxdt(11)= k(26)*(1-x(1))^3- k(225)*x(11);

%McRv

dxdt(12)= k(225)*x(11) - k(36)* x(12);

%Rv

dxdt(13)= k(3)*x(12) - 2* k(28)*x(13)*x(13) + 2*k(29)*x(15) - k(13)*x(13)+ k(14)*x(14) - k(32)*x(13);

%Rvn

dxdt(14)= - 2*k(38)*k(226)*x(14)*x(14) + 2*k(227)*x(16) + k(13)*x(13) - k(14)*x(14) - k(228)*x(14);

%RvRv

dxdt(15)= k(28)*x(13)*x(13) - k(29)*x(15) - k(178)*x(15) + k(191)*x(16) -2* k(229)*x(15);

%RvnRvn

dxdt(16)= k(38)* k(226)*x(14)*x(14) - k(227)*x(16) + k(178)*x(15) - k(191)*x(16) - 2* k(230)*x(16);

%Po

dxdt(17)=	k(1)*x(8) - k(9)*x(17)*C + k(10)*x(19) - k(5)*x(17);

%Pt

dxdt(18)= k(39)*x(10) - k(66)*x(18)*C + k(91)*x(20) - k(56)*x(18);

%PoC

dxdt(19)= k(9)*x(17)*C - k(10)*x(19) - k(16)*x(19) - k(57)*x(19);

%PtC

dxdt(20)= k(66)*x(18)*C - k(91)*x(20) - k(17)*x(20) - k(58)*x(20);

%PopC

dxdt(21)= k(16)*x(19) + k(67)*x(23)*C - k(92)*x(21) - k(4)*x(21) - k(6)*x(21) - k(179)*x(21) + k(192)*x(47) - k(11)*x(21)*x(45) + k(154)*x(37) - k(116)*x(21)*x(46) + k(156)*x(39);

%PtpC

dxdt(22)= k(17)*x(20) + k(68)*x(24)*C - k(93)*x(22) - k(41)*x(22) - k(18)*x(22) - k(180)*x(22) + k(193)*x(48) - k(117)*x(22)*x(45) + k(155)*x(38) - k(118)*x(22)*x(46) + k(157)*x(40);

%Pop

dxdt(23)= -k(67)*x(23)*C + k(92)*x(21) - k(42)*x(23) - k(119)*x(23)*x(45) + k(12)*x(27) - k(120)*x(23)*x(46) + k(148)*x(29) - k(181)*x(23) + k(194)*x(49);

%Ptp

dxdt(24)= -k(68)*x(24)*C + k(93)*x(22) - k(43)*x(24) - k(121)*x(24)*x(45) + k(147)*x(28) - k(122)*x(24)*x(46) + k(149)*x(30) - k(182)*x(24) + k(195)*x(50);

%PoppC

dxdt(25)= k(6)*x(21) - k(44)*x(25) + k(77)*x(35)*C -k(94)*x(25) + k(196)*x(51)- k(123)*x(25)*x(45) + k(158)*x(41) - k(124)*x(25)*x(46) + k(160)*x(43);

%PtppC

dxdt(26)= k(18)*x(22) - k(45)*x(26) + k(78)*x(36)*C -k(95)*x(26) + k(197)*x(52)- k(125)*x(26)*x(45) + k(159)*x(42) - k(126)*x(26)*x(46) + k(161)*x(44);

%PopRo

dxdt(27)= k(119)*x(23)*x(45) - k(12)*x(27) -k(69)*x(27)*C + k(96)*x(37) - k(183)*x(27) + k(198)*x(53);

%PtpRo

dxdt(28)= k(121)*x(24)*x(45) - k(147)*x(28) -k(70)*x(28)*C + k(97)*x(38) - k(184)*x(28) + k(199)*x(54);

%PopRt

dxdt(29)= k(120)*x(23)*x(46) - k(148)*x(29) -k(71)*x(29)*C + k(98)*x(39) - k(185)*x(29) + k(200)*x(55);

%PtpRt

dxdt(30)= k(122)*x(24)*x(46) - k(149)*x(30) -k(72)*x(30)*C + k(99)*x(40) - k(186)*x(30) + k(201)*x(56);

%PoppRo

dxdt(31)= k(127)*x(35)*x(45) - k(150)*x(31) -k(73)*x(31)*C + k(100)*x(41) + k(202)*x(57);

%PoppRt

dxdt(32)= k(128)*x(35)*x(46) - k(151)*x(32) -k(74)*x(32)*C + k(102)*x(43) + k(204)*x(59);

%PtppRo

dxdt(33)= k(129)*x(36)*x(45) - k(152)*x(33) -k(75)*x(33)*C + k(101)*x(42) + k(203)*x(58);

%PtppRt

dxdt(34)= k(130)*x(36)*x(46) - k(153)*x(34) -k(76)*x(34)*C + k(103)*x(44) + k(205)*x(60);

%Popp

dxdt(35) = - k(77)*x(35)*C + k(94)*x(25) + k(206)*x(61) - k(127)*x(35)*x(45) + k(150)*x(31) - k(128)*x(35)*x(46) + k(151)*x(32) - k(46)*x(35);

%Ptpp

dxdt(36) = - k(78)*x(36)*C + k(95)*x(26) + k(207)*x(62) - k(129)*x(36)*x(45) + k(152)*x(33) - k(130)*x(36)*x(46) + k(153)*x(34) - k(47)*x(36);

%PopCRo

dxdt(37) = k(11)*x(21)*x(45) - k(154)*x(37) + k(69)*x(27)*C - k(96)*x(37) - k(187)*x(37) + k(208)*x(63) - k(59)*x(37);

%PtpCRo

dxdt(38) = k(117)*x(22)*x(45) - k(155)*x(38) + k(70)*x(28)*C - k(97)*x(38) - k(188)*x(38) + k(209)*x(64) - k(217)*x(38);

%PopCRt

dxdt(39) = k(116)*x(21)*x(46) - k(156)*x(39) + k(71)*x(29)*C - k(98)*x(39) - k(189)*x(39) + k(210)*x(65) - k(60)*x(39);

%PtpCRt

dxdt(40) = k(118)*x(22)*x(46) - k(157)*x(40) + k(72)*x(30)*C - k(99)*x(40) - k(190)*x(40) + k(211)*x(66) - k(218)*x(40);

%PoppCRo

dxdt(41) = k(123)*x(25)*x(45) - k(158)*x(41) + k(73)*x(31)*C - k(100)*x(41) + k(212)*x(67) + k(59)*x(37);

%PtppCRo

dxdt(42) = k(125)*x(26)*x(45) - k(159)*x(42) + k(75)*x(33)*C - k(101)*x(42) + k(213)*x(68) + k(217)*x(38);

%PoppCRt

dxdt(43) = k(124)*x(25)*x(46) - k(160)*x(43) + k(74)*x(32)*C - k(102)*x(43) + k(214)*x(69) + k(60)*x(39);

%PtppCRt

dxdt(44) = k(126)*x(26)*x(46) - k(161)*x(44) + k(76)*x(34)*C - k(103)*x(44) + k(215)*x(70) + k(218)*x(40);

%Ro

dxdt(45) = - k(119)*x(45)*x(23) - k(127)*x(45)*x(35) - k(11)*x(45)*x(21) - k(123)*x(45)*x(25) + k(12)*x(27) + k(150)*x(31) + k(154)*x(37) + k(158)*x(41) - k(121)*x(45)*x(24) - k(129)*x(45)*x(36) - k(117)*x(45)*x(22) - k(125)*x(45)*x(26) + k(147)*x(28) + k(152)*x(33) + k(155)*x(38) + k(159)*x(42) + k(2)*x(4) - k(7)*x(45);

%Rt

dxdt(46) = - k(120)*x(46)*x(23) - k(128)*x(46)*x(35) - k(116)*x(46)*x(21) - k(124)*x(46)*x(25) + k(148)*x(29) + k(151)*x(32) + k(156)*x(39) + k(160)*x(43) - k(122)*x(46)*x(24) - k(130)*x(46)*x(36) - k(118)*x(46)*x(22) - k(126)*x(46)*x(26) + k(149)*x(30) + k(153)*x(34) + k(157)*x(40) + k(161)*x(44) + k(40)*x(6) - k(8)*x(46);

%PonpCn

dxdt(47) = k(79)* k(38)*x(49)*x(73) - k(104)*x(47) - k(216)*x(47) + k(179)*x(21) - k(192)*x(47) - k(131)* k(38)*x(47)*x(71) + k(170)*x(63) - k(132)* k(38)*x(47)*x(72) + k(172)*x(65) - k(48)*x(47);

%PtnpCn

dxdt(48) = k(80)* k(38)*x(50)*x(73) - k(105)*x(48) - k(219)*x(48) + k(180)*x(22) - k(193)*x(48) - k(133)* k(38)*x(48)*x(71) + k(171)*x(64) - k(134)* k(38)*x(48)*x(72) + k(173)*x(66) - k(49)*x(48);

%Ponp

dxdt(49) = - k(79)* k(38)*x(49)*x(73) + k(104)*x(47) - k(135)* k(38)*x(49)*x(71) + k(162)*x(53) - k(136)* k(38)*x(49)*x(72) + k(164)*x(55) + k(181)*x(23) - k(194)*x(49) - k(50)*x(49);

%Ptnp

dxdt(50) = - k(80)* k(38)*x(50)*x(73) + k(105)*x(48) - k(137)* k(38)*x(50)*x(71) + k(163)*x(54) - k(138)* k(38)*x(50)*x(72) + k(165)*x(56) + k(182)*x(24) - k(195)*x(50) - k(51)*x(50);

%PonppCn

dxdt(51) =	k(61)*x(47) + k(89)* k(38)*x(61)*x(73) - k(106)*x(51) - k(196)*x(51) - k(139)* k(38)*x(51)*x(71) + k(174)*x(67) - k(140)* k(38)*x(51)*x(72) + k(176)*x(69) - k(52)*x(51);

%PtnppCn

dxdt(52) = k(219)*x(48) + k(90)* k(38)*x(62)*x(73) - k(107)*x(52) - k(197)*x(52) - k(141)* k(38)*x(52)*x(71) + k(175)*x(68) - k(142)* k(38)*x(52)*x(72) + k(177)*x(70) - k(53)*x(52);

%PonpRon

dxdt(53) = k(135)* k(38)*x(49)*x(71) - k(162)*x(53) - k(81)* k(38)*x(53)*x(73) + k(108)*x(63) + k(183)*x(27) - k(198)*x(53);

%PtnpRon

dxdt(54) = k(137)* k(38)*x(50)*x(71) - k(163)*x(54) - k(82)* k(38)*x(54)*x(73) + k(109)*x(64) + k(184)*x(28) - k(199)*x(54);

%PonpRtn

dxdt(55) = k(136)* k(38)*x(49)*x(72) - k(164)*x(55) - k(83)* k(38)*x(55)*x(73) + k(110)*x(65) + k(185)*x(29) - k(200)*x(55);

%PtnpRtn

dxdt(56) = k(138)* k(38)*x(50)*x(72) - k(165)*x(56) - k(84)* k(38)*x(56)*x(73) + k(111)*x(66) + k(186)*x(30) - k(201)*x(56);

%PonppRon

dxdt(57) = k(143)* k(38)*x(61)*x(71) - k(166)*x(57) - k(85)* k(38)*x(57)*x(73) + k(112)*x(67) - k(202)*x(57);

%PtnppRtn

dxdt(58) = k(145)* k(38)*x(62)*x(71) - k(167)*x(58) - k(86)* k(38)*x(58)*x(73) + k(113)*x(68) - k(203)*x(58);

%PonppRtn

dxdt(59) = k(144)* k(38)*x(61)*x(72) - k(168)*x(59) - k(87)* k(38)*x(59)*x(73) + k(114)*x(69) - k(204)*x(59);

%PtnppRtn

dxdt(60) = k(146)* k(38)*x(62)*x(72) - k(169)*x(60) - k(88)* k(38)*x(60)*x(73) + k(115)*x(70) - k(205)*x(60);

%Ponpp

dxdt(61) = - k(89)* k(38)*x(61)*x(73) + k(106)*x(51) - k(206)*x(61) - k(143)* k(38)*x(61)*x(71) + k(166)*x(57) - k(144)* k(38)*x(61)*x(72) + k(168)*x(59) - k(54)*x(61);

%Ptnpp

dxdt(62) = - k(90)* k(38)*x(62)*x(73) + k(107)*x(52) - k(207)*x(62) - k(145)* k(38)*x(62)*x(71) + k(167)*x(58) - k(146)* k(38)*x(62)*x(72) + k(169)*x(60) - k(55)*x(62);

%PonpCnRon

dxdt(63) = k(131)* k(38)*x(47)*x(71) - k(170)*x(63) + k(81)* k(38)*x(53)*x(73) - k(108)*x(63) + k(187)*x(37) - k(208)*x(63) - k(62)*x(63);

%PtnpCnRon

dxdt(64) = k(133)* k(38)*x(48)*x(71) - k(171)*x(64) + k(82)* k(38)*x(54)*x(73) - k(109)*x(64) + k(188)*x(38) - k(209)*x(64) - k(220)*x(64);

%PonpCnRtn

dxdt(65) = k(132)* k(38)*x(47)*x(72) - k(172)*x(65) + k(83)* k(38)*x(55)*x(73) - k(110)*x(65) + k(189)*x(39) - k(210)*x(65) - k(63)*x(65);

%PtnpCnRtn

dxdt(66) = k(134)* k(38)*x(48)*x(72) - k(173)*x(66) + k(84)* k(38)*x(56)*x(73) - k(111)*x(66) + k(190)*x(40) - k(211)*x(66) - k(221)*x(66);

%PonppCnRon

dxdt(67) = k(139)* k(38)*x(51)*x(71) - k(174)*x(67) + k(85)* k(38)*x(57)*x(73) - k(112)*x(67) - k(212)*x(67) + k(62)*x(63);

%PtnppCnRon

dxdt(68) = k(141)* k(38)*x(52)*x(71) - k(175)*x(68) + k(86)* k(38)*x(58)*x(73) - k(113)*x(68) - k(213)*x(68) + k(220)*x(64);

%PonppCnRtn

dxdt(69) = k(140)* k(38)*x(51)*x(72) - k(176)*x(69) + k(87)* k(38)*x(59)*x(73) - k(114)*x(69) - k(214)*x(69) + k(63)*x(65);

%PtnppCnRtn

dxdt(70) = k(142)* k(38)*x(52)*x(72) - k(177)*x(70) + k(88)* k(38)*x(60)*x(73) - k(115)*x(70) - k(215)*x(70) + k(221)*x(66);

%Ron

dxdt(71) = - k(135)* k(38)*x(71)*x(49) - k(143)* k(38)*x(71)*x(61) - k(131)* k(38)*x(71)*x(47) - k(139)* k(38)*x(71)*x(51) + k(162)*x(53) + k(166)*x(57) + k(170)*x(63) + k(174)*x(67) - k(137)* k(38)*x(71)*x(50) - k(145)* k(38)*x(71)*x(62) - k(133)* k(38)*x(71)*x(48) - k(141)* k(38)*x(71)*x(52) + k(163)*x(54) + k(167)*x(58) + k(171)*x(64) + k(175)*x(68) - k(64)*x(71);

%Rtn

dxdt(72) = - k(136)* k(38)*x(72)*x(49) - k(144)* k(38)*x(72)*x(61) - k(132)* k(38)*x(72)*x(47) - k(140)* k(38)*x(72)*x(51) + k(164)*x(55) + k(168)*x(59) + k(172)*x(65) + k(176)*x(69) - k(138)* k(38)*x(72)*x(50) - k(146)* k(38)*x(72)*x(62) - k(134)* k(38)*x(72)*x(48) - k(142)* k(38)*x(72)*x(52) + k(165)*x(56) + k(169)*x(60) + k(173)*x(66) + k(177)*x(70) - k(65)*x(72);

%Cn

dxdt(73) = - k(79)* k(38)*x(73)*x(49) - k(89)* k(38)*x(73)*x(61) - k(81)* k(38)*x(73)*x(53) - k(85)* k(38)*x(73)*x(57) + k(104)*x(47) + k(106)*x(51) + k(108)*x(63) + k(112)*x(67) - k(80)* k(38)*x(73)*x(50) - k(90)* k(38)*x(73)*x(62) - k(82)* k(38)*x(73)*x(54) - k(86)* k(38)*x(73)*x(58) + k(105)*x(48) + k(107)*x(52) + k(109)*x(64) + k(113)*x(68) - k(83)* k(38)*x(73)*x(55) - k(87)* k(38)*x(73)*x(59) + k(110)*x(65) + k(114)*x(69) - k(84)* k(38)*x(73)*x(56) - k(88)* k(38)*x(73)*x(60) + k(111)*x(66) + k(115)*x(70) + k(48)*x(47) + k(52)*x(51) + k(49)*x(48) + k(53)*x(52);

%MnROR

dxdt(74) = k(232)*(1-x(1))^5 - k(233)*x(74);

%McROR

dxdt(75) = k(233)*x(74) - k(234)*x(75);

%ROR

dxdt(76) = k(235)*x(75) - k(236)*x(76) + k(237)*x(77) - k(238)*x(76);

%RORn

dxdt(77) = k(236)*x(76) - k(237)*x(77) - k(239)*x(77);

dxdt=dxdt';

return
